# Personality and Team Identification Predict Violent Intentions Among Soccer Supporters

**DOI:** 10.3389/fspor.2021.741277

**Published:** 2021-10-25

**Authors:** Joanna Lindström

**Affiliations:** Department of Psychology, Stockholm University, Stockholm, Sweden

**Keywords:** soccer violence, hooliganism, violent intentions, honesty-humility, team identification

## Abstract

Soccer supporter violence remains a persistent global problem. The majority of research examining the psychological underpinnings of soccer supporter violence have focused primarily on the role of team identification. Relatively little research has examined the role of basic personality traits and willingness to engage in violence amongst soccer supporters. In a study amongst Swedish soccer supporters (*N* = 247), we examined whether honesty-humility and team identification predict violent behavioral intentions; examining if collective narcissism mediates these associations. Honesty-humility negatively predicted violent intentions, and team identification predicted violent intentions. Collective narcissism partially mediated these associations. When both Honesty-humility and team identification are accounted for though, collective narcissism did not predict violent intentions. Such findings have implications for the design of violence prevention interventions amongst soccer supporters.

## Introduction

A resurgence in soccer supporter violence (e.g., Wheatstone, 2019; Hall, 2020), and recognition that such violence has become increasingly politicised (e.g., Brentin, 2018; Betz, 2020; Testa, 2020), reignites interest in revisiting what leads soccer supporters to engage in violence. A considerable amount of research has examined the role of identity-related processes, such as identification and identity fusion, in explaining why some soccer supporters resort to violence (Wann et al., 2003; Van Hiel et al., 2007; Newson et al., 2018). However, less research has examined the role of broad-band personality traits and engagement in soccer supporter violence. One personality trait which may underlie willingness to engage in violence in the soccer context, is honesty-humility from the HEXACO-PI (Ashton and Lee, 2009). Low honesty-humility manifests itself in narcissistic tendencies, and is associated with anti-social and aggressive behaviours (Allgaier et al., 2015; Thompson et al., 2016). Furthermore, a growing amount of research has shown that collective narcissism is related to hostile intergroup attitudes and behaviours (e.g., Golec de Zavala et al., 2013, 2016), suggesting that collective narcissism may also explain why some soccer supporters engage in violence for their team.

In the present research, we examined the relationship between honesty-humility and violent intentions amongst football supporters, and the relationship between soccer team identification and violent intentions. We further examined whether collective narcissism may play an important mediating role in these associations. In the following section we first examine the nature of violence which occurs amongst soccer supporters, subsequently reviewing the literature on social identification, honesty-humility, and individual and collective narcissism. Moreover, we examine how these variables may be interrelated, and may underlie willingness to engage in violence for one's soccer team.

Spectator violence is common amongst sports fans, but more prevalent amongst soccer supporters. Violence perpetrated by soccer supporters is a worldwide problem (Dunning, 2000), incurring numerous societal costs, stemming from death, injury, destruction of property, and police and security costs (Bridge, 2010). Soccer violence can take several forms. Some instances of violence may be considered as reactive forms of aggression, such as spontaneous brawls at soccer matches between supporters of opposing teams, whereas other forms of violence are planned, such as organised fights between soccer firms of opposing teams (Crouch, 2017). Historically, there has been a tendency to assume that individuals who engage in soccer-related violence (i.e., hooligans), are young, working-class men (Wakefield and Wann, 2006), or that soccer hooliganism is an expression of masculinity (Spaaij, 2008). However, soccer supporter violence does not solely occur amongst working class youth (Pearson and Stott, 2016).

The psychological research on soccer supporter violence has primarily focused on the role of team identification. According to Social Identification Theory (Tajfel and Turner, 1979), the ways that people think, feel and act, are highly shaped by the groups they identify with. When applying this perspective to sporting teams, team identification refers to the extent to which a team supporter/sports fan views their team as an extension of their self-identity. In other words, team identification can be conceptualised as the degree to which a fan/supporter feels a psychological connection to the team (Trail et al., 2000). Team identification and its various consequences have been studied extensively by researchers (e.g., Stott et al., 2010; Cikara et al., 2011). Researchers have examined the positive consequences of team identification, such as increased self-esteem and positive emotions (Branscombe and Wann, 1991). However, researchers have also drawn attention to the potential dark side of identification. Specifically, studies have shown that individuals who highly identify with their sporting team, are more inclined to engage in verbal and physical aggression towards supporters of opposing teams, or sporting officials (Wann et al., 2003; Porat, 2010).

However, such findings do not suggest that strong team identification automatically translates into aggressive or violent behaviours. Indeed, Wakefield and Wann (2006) identified two types of highly identified sports fans: those who engage in dysfunctional (i.e., antisocial) behaviours, and those who do not engage in such behaviours. Moreover, Wakefield and Wann (2006) suggested that the difference between these two groups is likely explained by individual differences.

Although it is recognised that personality predicts a range of different behaviours including interpersonal forms of aggression (see Bettencourt et al., 2006 for a review), when it comes to engagement in more *collective* forms of violence, researchers have been reluctant to focus on the role of personality. Some research has examined the role of more specific personality traits, such as rejection sensitivity and need to belong (Knapton et al., 2018). Other researchers have pointed to the potential role of “big five” personality traits, such as low agreeableness and conscientiousness (e.g., Van Hiel et al., 2007), though to the best of our knowledge, no research has examined the role of personality traits from the HEXACO personality inventory (HEXACO-PI; Ashton and Lee, 2009), a more updated measure of personality. One personality trait from the HEXACO-PI which may underlie soccer supporter violence is *honesty-humility*.

Honesty-humility is a prosocial personality trait, measuring the degree to which one is sincere, fair, modest, and greed avoidant (Ashton et al., 2014). Individuals scoring high on honesty-humility are not driven primarily by social status or economic incentives, and do not feel entitled to special treatment or privileges, viewing themselves as equals compared to others (Ashton et al., 2014). Conversely, individuals low in honesty-humility tend to be manipulative, and cheat, steal or deceive others for their own gain (Ashton et al., 2014). Individuals low in honesty-humility view themselves as superior compared to others, believing that they are entitled to special treatment (Ashton et al., 2014). They are also inclined to engage in antisocial behaviours, engaging in overt revenge (Thompson et al., 2016); acting aggressively in both provoked and unprovoked situations (Diníc and Smederevac, 2019). Individuals low in honesty-humility are also more inclined to choose to engage in (hypothetical) crimes (Van Gelder and De Vries, 2012). Given that honesty-humility is associated with antisocial and aggressive behaviours, and the aggressive and antisocial character of soccer-related violence, it is necessary to examine whether low honesty-humility amongst soccer supporters is associated with greater susceptibility to engaging in violence for one's team. To our knowledge, this has not previously been examined.

Conceptually, there is a great deal of overlap between honesty-humility and (individual) narcissism. A narcissist is an individual who has a grandiose self-image and a lack of empathy, and like individuals scoring low on honesty-humility, narcissists feel superior compared to others, considering themselves entitled to privileges. This often manifests itself in arrogant behaviour, and a need for constant attention and confirmation of one's own greatness (Bogart et al., 2004). Importantly, narcissists are invested in maintaining their grandiose self-perception, and tend to react aggressively in response to threats to their self-esteem, such as in the form of insults or criticism (Bushman and Baumeister, 1998). Given the conceptual overlap between honesty-humility and individual narcissism, could soccer supporters scoring low on honesty-humility, be more inclined to score highly on collective narcissism?

Collective narcissism refers to the belief that one's ingroup is exceptional, and hence deserving of privileged treatment, but not adequately recognised by others (Golec de Zavala et al., 2009). Specifically, collective narcissists tend to magnify perceived threats to their group, often feeling that their group is unfairly treated by their surroundings (Golec de Zavala et al., 2009). Indeed, a growing body of research has demonstrated that collective narcissism predicts a range of hostile attitudes and behaviours towards members of outgroups (see Golec de Zavala and Lantos, 2020 for a review). Specifically, collective narcissism is associated with the tendency to perceive the outgroup as hostile towards the ingroup (Golec de Zavala et al., 2019), and has even been found to predict support for terrorist violence (Jasko et al., 2020), and rejoicing in the suffering of outgroups (Golec de Zavala et al., 2016). Importantly, research also shows that collective narcissism predicts hostile retaliation towards other groups, when the ingroup's image has been undermined by outgroup members (Golec de Zavala et al., 2013).

Collective narcissism may be especially applicable to the soccer supporter context, since soccer supporters often believe that their team has been unfairly treated by the opposing team, sporting officials, or security and police. Furthermore, the image of a soccer supporters' team is often called into question by supporters of opposing teams, or following defeat during a soccer match (Branscombe and Wann, 1991). Furthermore, soccer hooligans who organise in “firms” often deem it essential to establish their status and reputation as the toughest compared to other firms. Members of such firms deem it important to stand up for one's team and fight against other firms, as well as against security and police officials. Specifically, mocking, provocations and other insults against the team or the firm's self-image are considered as triggering for soccer supporters (Spaaij, 2008).

Although research has shown that collective narcissism is associated with intergroup hostility in a number of intergroup settings, the majority of this research has focused on intergroup relations based on nationality, ethnicity, or religion (e.g., Golec de Zavala et al., 2009; Golec de Zavala and Cichocka, 2012). Although one study has examined the role of collective narcissism as moderating the association between team identification and dysfunctional fandom and aggression, amongst American football supporters (Larkin and Fink, 2019), no research has examined whether collective narcissism predicts violent intentions amongst soccer supporters, or whether collective narcissism may mediate the association between honesty-humility and violent intentions.

In summary, many soccer supporters identify strongly with their team, but do not resort to violence, suggesting the role of individual differences. The present research fills an important gap in the literature on soccer violence, by examining how honesty-humility, team identification and collective narcissism may be related to violent intentions amongst soccer supporters. In line with previous research (Wann et al., 2003; Porat, 2010), we expected a positive association between social identification and violent intentions (Hypothesis 1). Further, because honesty-humility negatively predicts anti-social and aggressive behaviours (Diníc and Smederevac, 2019), we expected a negative association between the honesty-humility and violent intentions (Hypothesis 2). Since individuals low in honesty-humility view themselves as superior to others; believing that they are entitled to special treatment, they may also be prone to experiencing collective narcissism. This suggests that collective narcissism could mediate the association between honesty-humility and violent intentions amongst soccer supporters. It is also reasonable to expect that a strong psychological connection to one's soccer team (i.e., team identification), may lead soccer supporters to view their team as exceptional, but not sufficiently recognised by others (i.e., collective narcissism). Thus, given significant associations between honesty humility and violent intentions, and social identification and violent intentions, we explored whether collective narcissism mediates the relationship between these associations.

## Method

### Participants

Participants consisted of 247 supporters of Hammarby football club (*M*_age_ = 37.93, *SD*_age_ = 13.27; 72.9% men). When examining educational attainment, 0.8% had not completed any formal education, 44.1% had completed high school, 17.8% had completed some post-high school education, 8.1% had completed courses at university (not resulting in a degree), 11.7% had a Bachelor's degree, and 9.3% had a postgraduate degree. With regard to socioeconomic status, 0.4% identified as upper class, 21.9 % identified as upper middle class, 58.3% identified as middle class, 12.1% identified as lower middle class, and 7.3% identified as working class. Hammarby supporters were recruited because they are the soccer club with the biggest supporter base in Sweden; and have the most hooligans according to some sources (Edwinsson, 2013).

### Procedure

Participants were recruited through several Facebook groups for Hammarby soccer supporters, and *via* snowball sampling. The study was advertised as a survey study examining experiences of Hammarby supporters and their attitudes towards violence. Inclusion criteria was identification as a Hammarby supporter and age of at least 18. Participants were compensated with a gift card (worth ~€5). This study was inspected by the National Ethics Board in Sweden, who deemed that there were no ethical problems with the study that required formal ethical approval (DNR: 2019-09-10).

This study was pre-registered, see https://osf.io/zcqpd/?view_only=fd6f854ba0a14c7fbd9415f21c9ba73c. Note that the main research questions and analyses outlined in our pre-registration are reported in another manuscript together with data from other several other studies sampling from non-sporting contexts (Lindström et al., manuscript in progress). Our examination of collective narcissism as a mediator was specified in this pre-registration as an exploratory analysis. We did not conduct an a priori power analysis, but aimed to have > 200 participants in line with general recommendations for sample sizes for structural equation modelling (e.g., Klein, 2011).

### Measures

The following variables were measured on a 7-point scale from 1(completely disagree) to 7(completely agree). Honesty-humility was measured with 10 items from the HEXACO Personality Inventory (Ashton and Lee, 2009; α = 0.73; e.g., “I think that I am entitled to more respect than the average person is”). Social (team) identification was measured with three items, adapted from earlier measures (Doosje et al., 1995; α = 0.74; e.g., “I feel a strong affinity with other Hammarby supporters”). Collective narcissism was measured with 7 items adapted from the Collective Narcissism Scale (Golec de Zavala et al., 2009; α = 0.78; e.g., “I am never going to feel satisfied until Hammarby gets all that we deserve”). Violent intention was measured with seven items adapted from previous measures (Doosje et al., 2013; Obaidi et al., 2019; α =0.90; e.g., “I am prepared to travel to other cities to fight for Hammarby”).

## Results

We first checked for multivariate outliers by computing Mahalanobis distances, detecting two cases of concern. Analyses with and without these two cases did not change the results, so we retained these cases. We also inspected descriptive statistics and correlations (see Table 1). As anticipated, honesty-humility was negatively correlated with violent intentions, social identification was positively correlated with violent intentions, collective narcissism was positively correlated with violent intentions, and honesty-humility was negatively correlated with collective narcissism. Inspection of collinearity diagnostics did not suggest that multicollinearity would be a concern.

**Table 1:** Descriptive statistics and correlations.

**Variable**	***M***	***SD***	**1**	**2**	**3**
Honesty-humility	4.68	0.78	–		
Team identification	6.13	0.83	−0.05	–	
Collective narcissism	4.66	1.07	−0.19^**^	0.42^**^	–
Violent intentions	3.02	1.56	−0.41^**^	0.28^**^	−0.26^**^

** *p < 0.01*.

To test our hypotheses, we conducted a series of regression analyses. We first regressed violent behavioural intentions simultaneously on team identification and honesty-humility. This model was statistically significant, *F*_(2, 211)_ = 35.85, *p* < 0.001, accounting for ~23.4% of the variance in violent intentions. Both team identification (β = 0.26, *p* < 0.001) and honesty-humility (β = −0.40, *p* < 0.001) were related to violent intentions, supporting both of our main hypotheses. Next, we regressed collective narcissism simultaneously on team identification and honesty-humility. This model was significant, *F*_(2, 222)_ = 28.39, *p* < 0.001, accounting for ~20.5% of the variance in collective narcissism. Further, both team identification (β = 0.55, *p* < 0.001) and honesty-humility (β = −0.19, *p* = 0.005) were related to collective narcissism.

Next, we tested a stepwise regression model where we included team identification and honesty-humility as predictors in the first step, and collective narcissism as a predictor in the second step. In the second step, collective narcissism only accounted for an additional 8% in the variance of violent intentions, and was not significantly related to violent intentions when identification and honesty-humility were included in the model (β = 0.10, *p* = 0.139).

To examine whether collective narcissism mediates the association between honesty-humility and violent intentions, and the association between identification and violent intentions, mediation analyses were conducted using Hayes (2017) method for mediation and the accompanying PROCESS custom dialogue. The 95% bias corrected confidence interval were based on 5,000 samples.

**Figure 1:**
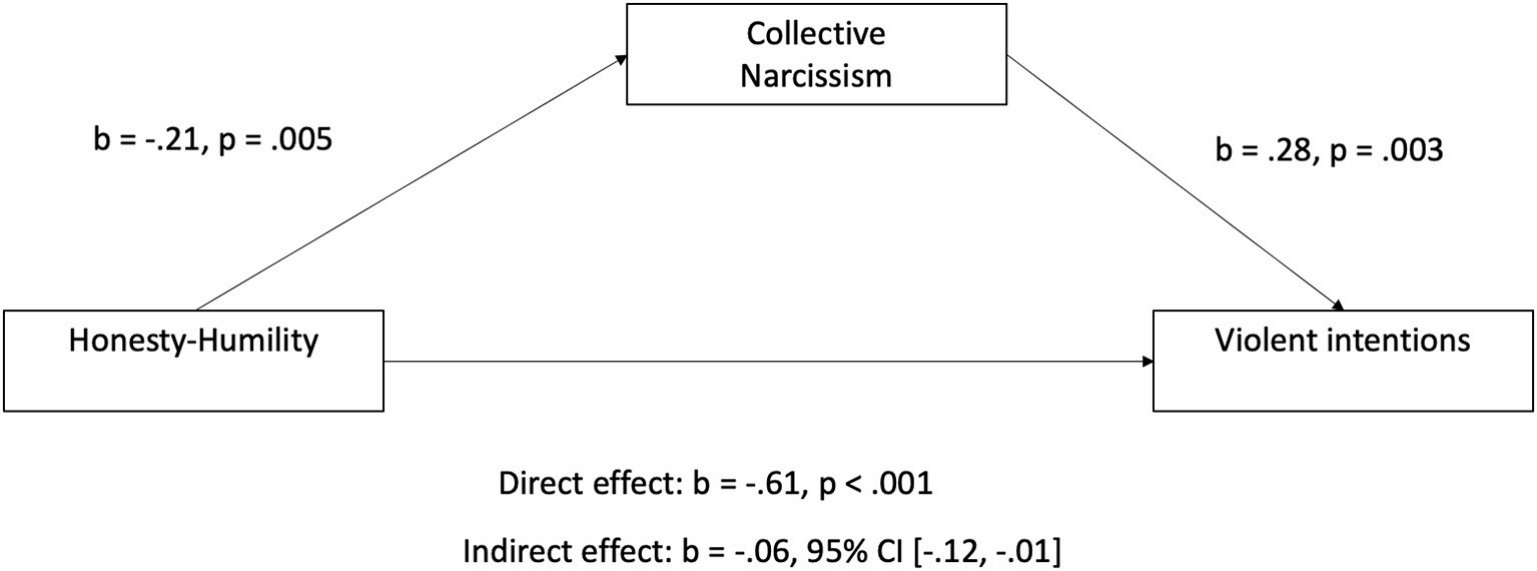
Mediation model of how collective narcissism partially mediates the association between honesty-humility and violent intentions amongst soccer supporters. Estimates are non-standardised estimates. Standardised estimates are presented in the text.

Since our original intention with examining the mediating relationships was not to examine mediation whilst controlling for the other variable, we first conducted two separate mediation analyses. Our first mediation model examined whether collective narcissism mediates the association between honesty-humility and violent intentions. In the following results we present standardised coefficients (see figures for unstandardised coefficients). Results of this analysis showed that honesty-humility negatively predicted collective narcissism (β = −0.19, *p* = 0.005), and collective narcissism positively predicted violent intentions (β = 0.19, *p* = 0.003). Furthermore, we observed an indirect effect of honesty-humility on violent intentions through collective narcissism [β = −0.04, 95% CI (−0.07, −0.01)]. However, we still observed a direct effect of honesty-humility on violent intentions [β = −0.37, *p* = < 0.001, 95% CI (−0.81, −0.40)], suggesting that collective narcissism may partially mediate the association between honesty-humility and violent intentions (see Figure 1).

**Figure 2:**
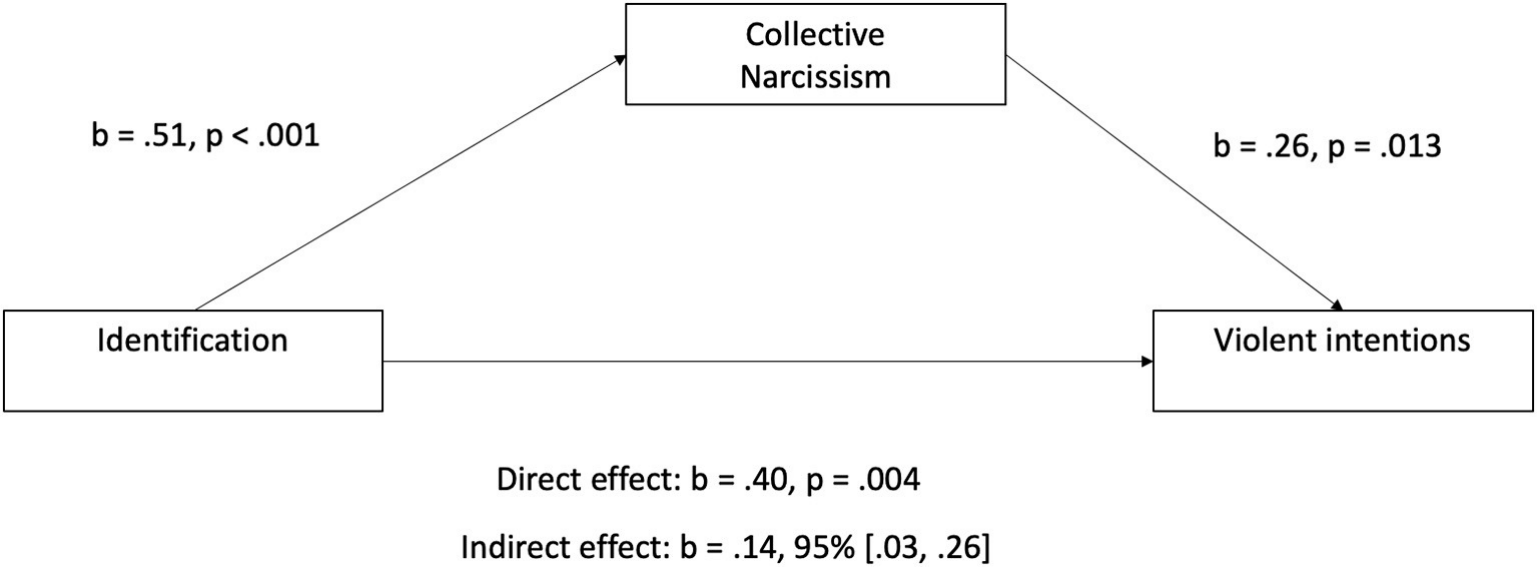
Mediation model of how collective narcissism partially mediates the association between identification and violent intentions amongst soccer supporters. Estimates are non-standardised estimates. Standardised estimates are presented in the text.

Our second mediation model examined whether collective narcissism mediates the association between identification and violent intentions. Results of this mediation analysis showed that identification predicted collective narcissism [b = 0.51, β = 0.39, *p* < 0.001, 95% CI (0.35, 0.67)], and collective narcissism positively predicted violent intentions [b = 0.26, β = 0.17, *p* = 0.013, 95% CI (0.06, 0.47)]. We observed an indirect effect of identification on violent intentions through collective narcissism, b = 0.14, 95% CI [0.03, 0.26]. We also observed a direct effect of social identification on violent intentions [b = 0.40, β = 0.20, *p* = 0.004, 95% CI (0.13, 0.67)], suggesting that collective narcissism may partially mediate the association between social identification and violent intentions (see Figure 2).

We conducted further analyses in Mplus (Muthén and Muthén, 2012). All models were estimated using Robust Maximum Likelihood (MLR) Estimation to deal with skewed variables and missing data, although to calculate indirect effects the model was estimated with Maximum Likelihood Estimation. Specifically, we tested an integrative model with both honesty-humility and identification as predictors, and collective narcissism as a mediator. This model was just-identified with 0 degrees of freedom. Honesty-humility negatively predicted violent intentions (β = −0.38, *p* < 0.001), and collective narcissism (β = −0.17, *p* = 0.005). Further, identification predicted violent intentions (ß = 0.22, *p* = 0.005), and collective narcissism (β = 0.41, *p* < 0.001), but collective narcissism did not significantly predict violent intentions (β = 0.10, *p* = 0.116) (see in Supplementary Figure 1 for path model).

**Figure 3:**
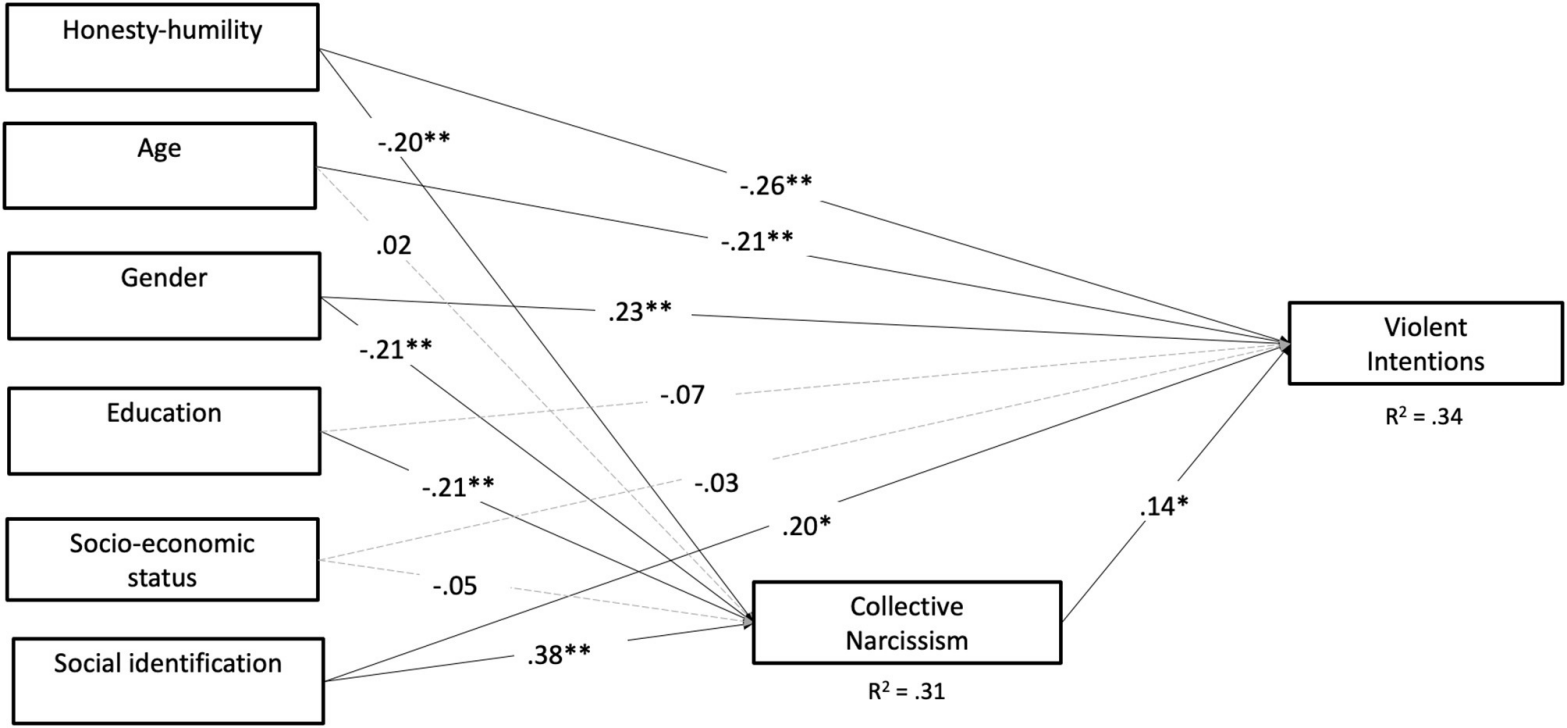
Path model predicting violent intentions amongst soccer supporters controlling for demographic variables. Estimates are standardised estimates. *p < 0.05 and **p < 0.01. Gender is coded: Women, 1 and Men, 2.

When we reran the above model controlling for demographic variables in the model, similar results were obtained but collective narcissism predicted violent intentions (β = 0.14, *p* = 0.03). See Figure 3 for path model controlling for demographic variables.

We also tested for indirect effects *via* collective narcissism by deriving 5,000 bootstrapped confidence intervals. The indirect effect of honesty-humility on violent intentions through collective narcissism was not significant [β = −0.03, 95% CI (−0.06,0.00)]. Neither was the indirect effect of identification on violent intentions through collective narcissism, [β = 0.05, 95% CI (−0.06, 0.00)].

## Discussion

In the present study we examined whether team identification and honesty-humility predicted violent intentions amongst soccer supporters, exploring whether collective narcissism may mediate these associations. Consistent with previous research linking team identification to self-reported verbal and physical aggression (Wann et al., 2003; Van Hiel et al., 2007; Porat, 2010), the results of our study showed that team identification predicted violent intentions. That is, individuals who identified strongly with their soccer team reported greater willingness to engage in violence for the sake of their team and other supporters.

Although researchers have argued that individual differences likely explain why some highly identified individuals engage in non-normative acts of fandom (e.g., Wakefield and Wann, 2006), relatively little research has examined whether individual differences in the form of basic personality traits, may explain soccer supporters' willingness to engage in violence. Our results demonstrated that willingness to engage in violence for one's soccer team, can indeed be explained by individual differences. Further, honesty-humility negatively predicted violent intentions amongst soccer supporters. Previous research has shown that individuals low in honesty-humility are more inclined to engage in interpersonal forms of aggression, such as overt revenge (Thompson et al., 2016), and provoked and unprovoked aggression (Diníc and Smederevac, 2019). Our findings suggest that low honesty-humility amongst soccer supporters may lead them to be more prone to engaging in violence for their team.

While research has shown that collective narcissism is associated with hostile attitudes towards outgroup members (Golec de Zavala et al., 2013; Golec de Zavala, 2019), the results of the present study suggest that the association between collective narcissism and violent intentions amongst soccer supporters, is less clear-cut. Collective narcissism partially mediated the association between honesty-humility and violent intentions, but not when statistically controlling for degree of team identification, and demographic variables. Similarly, collective narcissism partially mediated the association between team identification and violent intentions, but not when statistically controlling for honesty-humility, or demographic variables. Moreover, although collective narcissism was associated with violent intentions, when both team identification and honesty-humility are accounted for, collective narcissism does not predict violent intentions.

One explanation for this finding is that team identification (the extent to which a supporter feels a psychological connection to a team), and honesty-humility may both overlap to some extent with collective narcissism, but honesty-humility also captures behavioural tendencies to engage in antisocial behaviour. As such, when both of these variables are accounted for, collective narcissism as a predictor of violent intentions becomes redundant. Furthermore, the results demonstrated an association between honesty-humility and collective narcissism, implying that increasing honesty-humility may both decrease collective narcissism and violent intentions amongst soccer supporters. While honesty-humility is a disposition which may be difficult to change, research shows that experimentally inducing (state-based) humility reduces motivation to aggress (Summerell et al., 2020), suggesting that this may be a promising method for violence prevention interventions.

### Limitations and Directions for Future Research

The present study suffers from some limitations. The present study utilised a correlational design and therefore causality cannot be established. Although we tested mediation models based on existing theory and research, mediation models based on correlational data not provide sufficient evidence of causality (Bullock et al., 2010). Furthermore, we collected data from supporters of one soccer team in Sweden, which may limit generalisability to other team settings. Future research should conduct longitudinal studies amongst soccer supporters, as well as supporters of different sporting teams.

An additional limitation was that the violent behavioural intention measure did not specify targets of violence. Although our intention was to keep the scale as similar as possible to previous measures (e.g., Doosje et al., 2013; Obaidi et al., 2019), it is possible that results may differ according to the targets of violence. Importantly, given the recent increase in soccer supporter violence with political agendas, such as the rise of neo-Nazi soccer hooligans (see Parkin, 2018), future research should extend the results of our present study by measuring soccer supporters' political orientation, and incorporating different targets of violence (i.e., supporters of rival teams, sporting officials, police, ethnic or religious minority groups) in violent intention measures. Such research could examine similarities and differences between different sub-types of soccer hooligans (i.e., politically-motivated soccer hooligans vs. “traditional” hooligans who are not motivated by politics), and whether psychological predictors of soccer violence differ according to the target of violence.

### Implications and Conclusion

The findings of the present study suggest that soccer supporters scoring low in honesty-humility and high in team identification may be especially susceptible to engaging in violence for their team. Low honesty-humility and high team identification may increase soccer supporters' willingness to engage in violence for their team. The results of the present study provide some preliminary evidence to suggest that soccer violence prevention programmes targeting highly identified supporters, may be more effective in reducing soccer-related violence, if they also encourage soccer supporters to feel humility.

## Data Availability Statement

The datasets presented in this study can be found in online repositories. The names of the repository/repositories and accession number(s) can be found below: 10.17045/sthlmuni.14980251.

## Ethics Statement

Ethical review and approval was not required for the study on human participants in accordance with the local legislation and institutional requirements. The patients/participants provided their written informed consent to participate in this study.

## Author Contributions

JL designed the study and survey, prepared ethics application, obtained funding, collected the data, analysed the data, and wrote the manuscript.

## Funding

This study was supported by a grant to JL by Lars Hierta's Memorial Foundation (FO2019-0005).

## Conflict of Interest

The author declares that the research was conducted in the absence of any commercial or financial relationships that could be construed as a potential conflict of interest.

## Publisher's Note

All claims expressed in this article are solely those of the authors and do not necessarily represent those of their affiliated organizations, or those of the publisher, the editors and the reviewers. Any product that may be evaluated in this article, or claim that may be made by its manufacturer, is not guaranteed or endorsed by the publisher.
