# Supplementary figures and images for: Personality and Team Identification Predict Violent Intentions Among Soccer Supporters
Source: Front Sports Act Living. 2021 Oct 25;3:741277. doi: 10.3389/fspor.2021.741277 (PMC8573121; doi:10.3389/fspor.2021.741277)

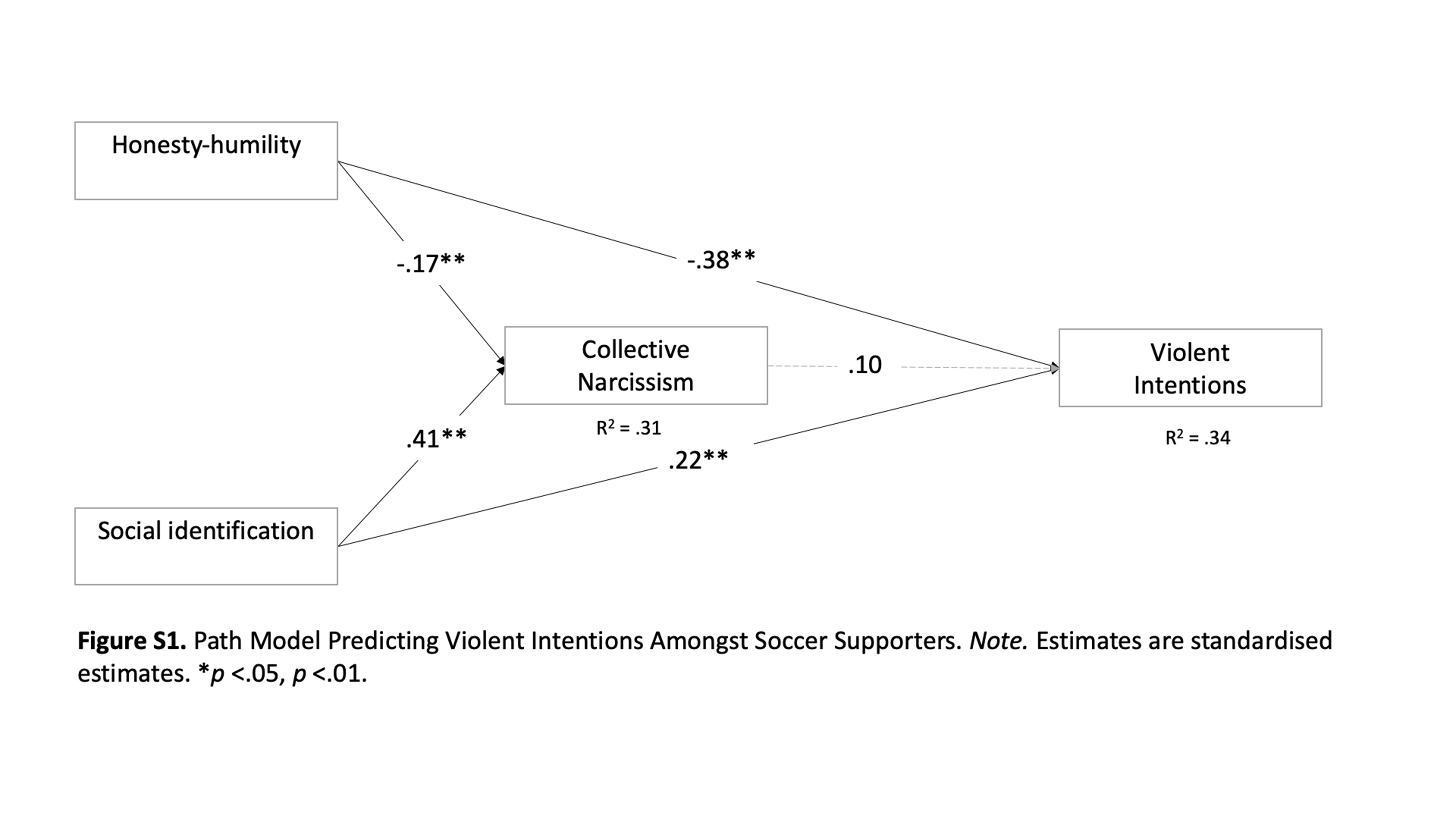

Supplement: Supplementary file 1 [file Image_1.tiff]
